# Inclusive classroom climate development as the cornerstone of inclusive school building: review and perspectives

**DOI:** 10.3389/fpsyg.2023.1171204

**Published:** 2023-09-01

**Authors:** Nicolas Margas

**Affiliations:** Institute of Sport Sciences, University of Lausanne, Lausanne, Switzerland

**Keywords:** inclusion, classroom environment, inclusive teaching, special education needs (SEN) students, integration, universal teaching

## Abstract

Education institutional guidelines around the world agree that building more inclusive schools is a priority. The reality of school practice, however, belies this institutional will. To help fill the gap, this theoretical review documents the value that the construct of classroom climate brings to research and practice in terms of inclusive school development. The article firstly points out that the current main challenge is to develop Inclusive Mainstream Teaching (IMT) in diverse classrooms. Indeed, IMT is needed in all classrooms to guarantee the effectiveness of special accomodating measures in schools that are targeted at special education needs students. Intervening at classroom level is both a pragmatic and powerful way of developing inclusive schooling. However, developing IMT in the classroom remains a challenge for both teachers and researchers. Thus this review documents the central role that classroom climate should play in the development of IMT. More precisely, the factors of classroom climate that are associated with inclusive outcomes are identified. We also highlight how these factors and the measurements associated with them are efficient tools to guide IMT development. These measures are proximal, sensitive, complementary, and pragmatic indicators of effective IMT. Such indicators are very useful in helping research empirically document effective IMT, ensure that any small improvement is assessed, monitor teachers’ progress, and assist their professional growth. Theoretically positioned as a mediator between inclusive teaching in mainstream classrooms and inclusive school outcomes, inclusive classroom climate is a tool that appears to be effective in supporting IMT development and, consequently, in the establishment of more inclusive schools.

## 1. Introduction

Building more inclusive schools is a core element of international educational guidelines (UNESCO, 2015, 2016) and school policies around the world (Peters, 2004; Curcic, 2009; Katz, 2013; Watkins, 2017; Schwab, 2021). This political and institutional consensus is accompanied by a shared definition of the goals of inclusive education (Avramidis et al., 2000; UNESCO, 2009; Katz, 2013). The “ultimate” goal of inclusive education (Booth and Ainscow, 2002; Schwab, 2021) is that school forms “the basis for a just and non-discriminatory society” (UNESCO, 2009, p. 9). As such, this is a deeply social goal. Inclusive schools must promote positive relationships between diverse students, peer acceptance and a sense of community for all, including those with special education needs (SEN; Koster et al., 2009; Bossaert et al., 2013). Since this first objective can only be achieved in a context of diversity, the second, more academic objective, is engagement and learning for all students despite the differences between them. Striving to achieve these goals is also crucial for overcoming other recurrent educational issues like bullying (e.g., Thornberg et al., 2022), school dropout (e.g., Reinke and Herman, 2002), students’ well-being (e.g., Wang et al., 2020b) and even long-term health (e.g., Holt-Lunstad et al., 2015).

However, despite agreement over the objectives of an inclusive school, developing inclusive practices remains a major challenge (Ferguson, 2008; Heiniger and Hercod, 2017). The philosophical underpinnings of inclusive education are generally shared by teachers (Jury et al., 2021), but difficulties remain when it comes to implementation. This leads to skepticism, lack of confidence if not outright rejection by teachers of the inclusive approach (Desombre et al., 2019; Savolainen et al., 2022; Jury et al., 2023).

This theoretical review argues that there is a need to draw on the construct of classroom climate to overcome the gap between institutional objectives and the reality of the classroom. It followed a three-steps argumentation^1^. The first section point out that a top priority is to develop inclusive mainstream teaching (IMT) in diverse classrooms. Next, the article documents the central role that the classroom climate can play in developing IMT. The second section identifies the factors of the classroom climate that produce inclusive outcomes. The third section shows how those factors of inclusive classroom climate and the measures associated with them are tools that can efficiently support IMT development in classrooms.

## 2. Inclusive mainstream teaching as the current main challenge to developing inclusive schools

Although segregated schools for students with special education needs (SEN) still exist, most educational systems around the world aim to welcome as many students as possible in the same schools (for reviews, Curcic, 2009; Schwab, 2021). Nearly all the enrolled school population is now educated in mainstream schools (e.g., 97.36% in Education, E.A.f.S.N.a.I, 2017). To adapt these schools to such diversity, guidelines propose a variety of special accommodations targeting certain students around a mainstream form of teaching that is required to be more inclusive (Schwab, 2021). “Special accommodations” refer to the permanent or temporary formation of special needs groups within the school and to special needs teaching that occurs within or alongside mainstream classes. Such accommodations generally concern about 15% of school pupils, including pupils with learning or/and behavioral difficulties at school and students with officially-recognized specific needs (4.53% of students in Europe; Education, E.A.f.S.N.a.I, 2017). IMT is intended to provide the opportunity to as many students as possible to be included in mainstream teaching. The social aim is to create connections between diverse students and the pedagogic aim is to be flexible and differentiated enough to offer the best possibilities for development and learning for all (Willis and Mann, 2000; Tomlinson et al., 2003). The following empirical findings show that generalizing the development of IMT to all classrooms is what is currently needed to establish inclusive schools.

### 2.1. Inclusive mainstream teaching and efficiency of special accommodations

In this section, we review empirical evidence showing that special accommodations for SEN struggle to be efficient if they are not articulated with IMT in all classrooms.

First of all, special accommodations that permanently segregate students lead to mixed results in terms of academic achievement (Chiu et al., 2017). Moreover, this segregation gives no opportunity for cross-group interactions, thus doing nothing to counter stereotype-ridden, negative attitudes towards SEN students among the mainstream pupil population (for a review, Juvonen et al., 2019). Intergroup contacts in schools are necessary to enhance positive attitudes and trust towards minorities (Hewstone et al., 2018), cross-ethnic friendships, the emergence of complex social identities (Knifsend and Juvonen, 2017) and more positive outgroup stereotypes (Munniksma et al., 2013). In segregated situations, precautions are needed to ensure that intergroup contacts outside the classroom are constructive and even sought after. This requires the development of inclusive education principles in all students, i.e., an efficient IMT. Indeed, inclusive peer norms, low intergroup anxiety, expectation of similarity, the valuing of difference, social competencies like perspective taking, empathy and tolerance, are all essential to prompt mainstream students to be open to mixing with SEN students (for a review, Turner and Cameron, 2016). In sum, IMT is needed to encourage intergroup contact with SEN students and reduce the exclusion effects of special accommodations that isolate them.

In the second case, where special accommodations are based on the creation of temporary special groups and specialized interventions within mainstream classrooms, intergroup contacts do exist. Such arrangements are common in many countries. For example, most SEN students are placed in mainstream classrooms for 80% or more of their time in European schools (UNESCO, 2016). However, such settings can also lead to stereotype-reinforcement and status differences (Bigler and Liben, 2007). Without IMT, this intergroup saliency can actually reinforce discrimination and exclusion (Córdova and Cervantes, 2010; Covelli and de Anna, 2020). For example, special accommodations targeted at SEN can at times be seen as an unfair use of resources by mainstream students—especially those experiencing academic difficulty, their parents and even teachers. In such circumstances, even more discrimination may result, if SEN students are perceived as a threat to mainstream students’ development and to values of meritocracy (for reviews, Iyer, 2022; Stanczak et al., 2023). Similarly, if achievement is defined in terms of superiority over others in the classroom (i.e., normative comparison), special accommodations can result in SEN students again being seen as a threat to mainstream students’ achievements. This can lead to rejection of SEN students and their feelings of exclusion (Iyer, 2022).

Interestingly, some studies show a third scenario. In this case, offering special accommodations initially provided just for students with SEN, to other mainstream students when necessary, can contribute to breaking down intergroup divisions and reducing threat perceptions. For example, high-quality co-teaching between mainstream and specialized teachers within an inclusive setting facilitates learning for students with and without SEN, social acceptance of students with SEN and socio-emotional development for all (Bear and Proctor, 1990; Juvonen and Bear, 1992; Schwab, 2017).

Taken together, these findings highlight that mainstream and special teaching needs to be more inclusive in all contexts (Hornby, 2015). IMT development is thus a key to the development of truly inclusive schools.

### 2.2. Classroom teaching as a pragmatic and powerful element to develop inclusive schools

IMT within a classroom is a considerable challenge, but less than developing an entire inclusive school. IMT is circumscribed to the classroom environment specifically cultivated by one teacher (Schweig et al., 2019). Moreover, modifying the way to teach in their classroom is the teacher’s foremost concern. This is the element in which they will invest the most (Bonvin and Margas, 2021). Seeking to build an entire inclusive school requires both training and convincing virtually every member of the educative team. Since training courses are often organized by academic discipline, this is rarely the case. Inversely, even isolated teachers can seek to implement IMT. This perspective fits with the relative independency of each classroom dynamics in a school (Wang and Degol, 2016). The progress of one teacher in implementing IMT may even give others self-confidence and trigger a broader transformation.

The classroom level is not just more pragmatic for developing inclusive schools. It is also the main source of variation in students’ learning and achievement (Nye et al., 2004; Pianta and Hamre, 2009). The classroom is the environment in which students learn and interact with peers and teachers on a daily basis (Brackett et al., 2012; Fraser, 2015). These daily interactions are the primary processes that provide students the opportunity to develop academic and social competencies (Hamre, 2014). As such, the development of IMT in classrooms seems the most pragmatic and efficient factor for developing inclusive schools.

### 2.3. Inclusive mainstream teaching: the main current issue for teachers and researchers

Special accommodations are often explicitly defined in institutional directives because they are often associated with specific fundings (Schwab, 2021) and because they build on the legacy of previous special education development efforts (Bedoin and Séguillon, 2021). However, this is not the case for IMT. Institutional directives focus only on the objectives of IMT and, at best, describe what IMT needs to develop in an appropriate way (e.g., differentiated instruction, accessible teaching content, positive relationships between students in classrooms, cooperation) without indicating how. The implementation of IMT is therefore left to teachers who can feel powerless and helpless. As evidence of this situation, many teachers express concerns about inclusive education and more precisely, by order of importance, (a) the lack of resources in terms of staff and funding, (b) the extended working hours induced by an inclusive classroom, (c) the difficulties associated with IMT, and (d) the appropriateness of inclusive education in the classroom, which may lead to reduced learning for mainstream students (Jury et al., 2023). This last issue appears the most frequently in teachers’ concerns about inclusive education (Jury et al., 2023). Teachers’ negative attitudes are also often fueled by a perceived lack of self-efficacy (Desombre et al., 2019) and the feeling that they are unable to cope with specific students’ difficulties (de Boer et al., 2011; Monsen et al., 2014). In sum, the main issue behind teachers’ resistance to developing IMT is the perceived difficulty of teaching in a diverse classroom.

It is not very surprising that educational guidelines are unclear about IMT implementation and that teachers are reluctant to try, considering that even research has trouble identifying effective IMT strategies (Juvonen et al., 2019; Schwab, 2021). Empirical evidence of successful IMT implementation is largely lacking, especially in contexts of diversity (Bossaert et al., 2012; De Vroey et al., 2016; Loreman, 2017; Fabes et al., 2019). For example, the development of differentiated instruction leads to diverse practices, with no consensus or empirical conclusions to guide these practices (Galand, 2017). Similarly, the management of social dynamics in the classroom lacks empirical evidence (Farmer et al., 2018). These observations help us to understand the lack of existing teacher training on IMT (Stough, 2006; Webster-Stratton et al., 2008). For many authors (Juvonen et al., 2019; Van Mieghem et al., 2020) and politicians (United Nations Human Rights Special Procedure, 2019), the key foundation for successful implementation of IMT remains the development of evidence-based trainings. Yet, identifying empirically validated IMT practices constitutes the most prominent challenge to developing inclusive schools, according to teachers, researchers and even politicians. In other words, how do we know if the IMT used actually make a difference? As IMT obviously works at the level of the classroom, taking classroom climate into consideration seems a particularly promising way of testing its efficacy. The two following sections present the model of classroom climate and evidence that classroom climate represents an effective approach for developing IMT.

## 3. Classroom climate and the achievement of inclusive school goals

Coming from organizational psychology, the concept of climate refers to the feel of an environment (e.g., the school, the classroom) that emerges from actors’ perceptions of their experiences in this environment (e.g., Ostroff et al., 2003). Even though these experiences may vary from one day to another, they converge towards “a consistent image of the long-standing attributes of classroom environment” (Fraser, 1998, p. 8). Classroom climate thus corresponds to the overall perception of relatively stable characteristics and social interactions that occur within the classroom environment (Filiault and Fortin, 2011).

A major finding on classroom climate is that students’ perceptions of their experiences in the classroom are critical in guiding their behaviors and, consequently, their engagement, learning and social behaviors at school (Fraser, 1998; Wang, 2012; Wagner et al., 2013; Wang et al., 2020b). Students’ perceptions of different factors of classroom climate are thus key to understanding how modifying teaching in the classroom may affect both goals of inclusive schooling.

### 3.1. Classroom climate multidimensional model

Conceptualizations of classroom climate encompass the different processes experienced in classrooms (for reviews, Hamre et al., 2007; Downer et al., 2010; Kaplan Toren and Seginer, 2015). As classroom climate refers to the perception of the classroom environment stemming from various types of experiences, classroom climate models are multidimensional. Following Moos and Trickett (1974) and Walberg and Anderson (1968) conceptualizations, these models all converge towards at least three basic dimensions (Fraser, 1998; Pianta and Hamre, 2009; Filiault and Fortin, 2011; Fauth et al., 2014; Bardach et al., 2020; Wang et al., 2020b), even if the terminologies and boundaries of these dimensions sometimes diverge.

The first dimension, often called the *relationship* (Fraser, 1998) or *socioemotional support* dimension (Wang et al., 2020b) refers to the perceptions of the “nature and intensity of personal relationships within the environment and assesses the extent to which people are involved in the environment and support and help each other” (Fraser, 1998, p. 9). It relies on the social and emotional wellbeing of students, including the warmth, safety, connectedness, quality of interactions between teachers and peers, and their consecutive sense of belonging to the classroom (Filiault and Fortin, 2011; Wang et al., 2020b). The second dimension is named the *system maintenance and change dimension* (Fraser, 1998) or *organization and management dimension* (Wang et al., 2020b). It includes perceptions of the organization inside the classroom such as clarity of rules and order, openness to negotiation. This dimension is related to the management of students’ behavior, time, and attention in the classroom (Hamre et al., 2007). Finally, the *personal development* (Fraser, 1998) or *instructional dimension* (Wang et al., 2020b) assesses the perceptions of instruction strategies and learning processes, which favor (or not) students’ personal growth and learning in the classroom (Fraser, 1998). This dimension is dependent on supportive interactions that facilitate learning, the provision of challenging tasks, and constructive feedback (Hmelo-Silver et al., 2007; Fauth et al., 2014).

A construct validity approach suggests that theory, measurement, empirical research, and practice are intertwined and that the neglect of one aspect can undermine the others and the resulting validity of the construct (Marsh, 2002). Such an approach is useful in appreciating the relevance of the classroom climate model. When it comes to the factors within the three basic dimensions of classroom climate, the relations between theory, measurement and empirical results are well-documented (for reviews, Fraser, 1998; Pianta and Hamre, 2009). Recent meta-analysis (Wang et al., 2020b) and large scale studies (e.g., Hamre et al., 2014) showed relations between these factors of classroom climate and important educational outcomes. Moreover, beyond the validated scales that exist to measure each specific factors of classroom climate, some measurement instruments regrouped several of these scales (for reviews, Fraser, 1998; Altaf, 2015; Fraser, 2015) and revealed good factorial validity (e.g., the WIHIC, Skordi and Fraser, 2019). Adaptations of these instruments for various types of schools and students exist (e.g., Beld et al., 2018). Researchers can therefore choose the appropriate instrument or even part of this instrument to test their hypothesis in various contexts. It is even assumed that “few fields in education can boast the existence of such a rich array of validated and robust instruments that have been used in so many research applications” (Fraser, 1998, p. 8).

Despite this solidity, more research is needed to specify the exact definition and boundaries of the three basic dimensions of classroom climate. The terminologies of these dimensions vary and the specific factors included in those dimensions can also vary from one model to another (see, Fraser, 1998; Pianta and Hamre, 2009). For example, perceived autonomy support in the classroom may, depending on how it is conceptualized, be included in the *relationship* or *socio-emotional dimension* (Pianta and Hamre, 2009), the *organization and management dimension* (Wang et al., 2020b), or the *instructional* or *personal development dimension* (Moos and Trickett, 1974). Similarly, the perception of safety in the classroom (i.e., physical and emotional security) is proposed as a specific fourth dimension (Wang and Degol, 2016), included in the *relationship dimension* or even the *instructional dimension* (e.g., Wang et al., 2020b). To add to the confusion, empirical tests of the three dimensions model of classroom climate are not yet very conclusive (e.g., Hamre et al., 2014).

Nevertheless, in practice, researchers rarely need to encompass the classroom climate as a whole. They often only choose the appropriate validated scales depending on the specific factors of classroom climate they are focusing on and their hypotheses (Fraser, 2015). In sum, the issue of the precise definition of the three basic dimensions of classroom climate does not prevent researchers from identifying the antecedents and consequences of specific factors of classroom climate. These factors of classroom climate, whatever the dimension they belong to, are theoretically and empirically posited as mediators between classroom teaching and important educational outcomes and can be measured with validated instruments. They represent a promising perspective to identify key elements of IMT. The next section hence reviews empirical work identifying factors of classroom climate that help accomplish the social and academic goals of inclusive schools.

### 3.2. The relations between factors of classroom climate and inclusive outcomes

The social objective of an inclusive school is to improve social behaviors between peers, especially that involving potentially stigmatized peers. The academic objective of an inclusive school is to promote engagement, learning and achievement for all students, particularly those with learning difficulties, in a context of diversity. This section reviews factors of classroom climate that are associated with such outcomes. These are presented according to the three proposed basic dimensions of classroom climate, bearing in mind that these dimensions are open to discussion. The objective here is to focus on factors of classroom climate that are related to inclusive outcomes.

Concerning the *relationship dimension* of classroom climate, previous results have identified three factors that foster achievement in terms of both the social and academic goals that inclusive schools aim for. Firstly, perceived quality of relations between classroom peers and perceived quality of relations between pupils and teachers are two important factors of an inclusive classroom climate and they are associated with both social and academic goal fulfilment. More precisely, these two aspects of the relational classroom climate are related to students’ social behaviors (Kellam et al., 1998; Mooij, 1999; Roubinov et al., 2020), especially peer victimization and bullying (Barboza et al., 2009; Gregory et al., 2010; Raskauskas et al., 2010; Gendron et al., 2011; Turner et al., 2014; Thornberg et al., 2017; Gage, 2020; Montero-Montero et al., 2021). Perceived quality of relations between peers and between pupils and teachers are also related to students’ engagement, self-determination, efficient learning self-regulation and achievement (Ferguson and Dorman, 2003; Anderson et al., 2004; Lynch and Soukup Sr, 2017). Meta-analyses have found moderate relationships between the quality of teacher-students relations and students’ engagement at school and achievement (e.g., Quin, 2017), as well as general executive functioning (Vandenbroucke et al., 2018). Additionally, longitudinal large cross-sectional studies reveal the short-term and long-term benefits of positive peer relations and pupil-teacher relations in terms of reduced aggression and exclusion between students, and also students’ academic results (e.g., Avant et al., 2011; Thornberg et al., 2022). Crucially from the point of inclusion, even if all children benefit from the quality of relationships in the classroom, this is especially true for at-risk, stigmatized, and vulnerable students. Indeed, the positive effects of the quality of classroom relations on such students in terms of reduced exclusion, improved social adjustment, less deviant peer affiliation and greater sense of belonging within the school (Gazelle, 2006; Avant et al., 2011) have been well documented. Furthermore, their active academic engagement and achievement are also improved (Hamre and Pianta, 2005; Berti et al., 2010; Allodi, 2010a). The third factor of an inclusive classroom climate is the perceived belonging to the classroom (Bossaert et al., 2013). Perceived classroom belonging is related to the academic self-efficacy, intrinsic motivation and task value perceived by students and their sense of belonging and social acceptance (Freeman et al., 2007). It is also related to degree of persistence for African American undergraduate women (Booker, 2016). In sum, students’ sense of classroom belonging, student-student and student-teacher relationships in the classroom are three crucial factors in achieving both the social and academic objectives of inclusive schools. Yet they are often neglected in educational policies and teacher training programs (Allodi, 2010b).

Concerning the *organization and management dimension* of the classroom climate, four factors appear to be linked to the two desired outcomes. First of all, a meta-analysis has showed that the perceived quality of class organization and clarity of classroom rules improve behavioral and emotional outcomes (Korpershoek et al., 2016), as well as social competence (Shechtman, 2006; Barbarin et al., 2010). Moreover, these factors also lead to higher math and reading achievements over time (Bennacer, 2000; Ponitz et al., 2009; Gaskins et al., 2012; Hatfield et al., 2016; Korpershoek et al., 2016) and more efficient self-regulation (Brody et al., 2002), engagement and task persistence in the classroom (Bohn et al., 2004; Rimm-Kaufman et al., 2009). Even more important for effective inclusion, the quality of classroom organization in an inclusive setting is associated with social and academic outcomes for special needs students as well as for others (Ainscow et al., 2006). Students’ autonomy management is a second factor of the organization and management dimension which helps achieve inclusion-related social and academic objectives. Indeed, the perceived authoritarianism of teachers is directly related to bullying (Thornberg, 2018) and, inversely, a democratic management climate in the classroom promotes democratic values in students and attitudes of responsibilities inside as well as outside the school (Torney-Purta et al., 2001). Complementarily, a student-centered style of teaching (i.e., where students are considered to be active participants of their learning and where teachers try to facilitate students’ autonomous efforts to learn) induces positive social consequences for students susceptible to stress whilst having no negative impact on less sensitive students (Roubinov et al., 2020). A third related inclusive classroom climate factor resides in the feeling of justice (or injustice) between students in the classroom. More precisely, feelings of injustice from teacher management are negatively associated with academic self-efficacy (Dorman, 2001), engagement (Berti et al., 2010), learning motivation (Dalbert and Maes, 2002), and even long-term academic achievement (Resh and Sabbagh, 2009). Feelings of injustice, moreover, modify the conception of equity and just societies (Dar and Resh, 2003). Finally, the fourth factor observed in the literature refers to the social norms that are perceived as salient in the classroom. Indeed, salient pro-inclusion social norms in the classroom lead to more inclusive behaviors in all students, a stronger sense of belonging among students from marginalized groups, and a reduction in the achievement gap (Murrar et al., 2020; Brauer et al., 2021).

Examining the *instructional dimension* of classroom climate, we find three factors related to inclusive outcomes. Firstly, the perceived differentiation of learning improves social participation in the classroom (Zurbriggen et al., 2021) and achievement (Fast et al., 2010; Curby et al., 2011), a positive effect that applies to at-risk students as well (Hamre and Pianta, 2005). Conversely, lack of consideration for differences between pupils is associated with school disengagement and absenteeism (Fallis and Opotow, 2003). Secondly, a climate that supports pupil perceived autonomy and avoids perceptions of teacher control leads to prosocial behavior between students (Cheon et al., 2019). And thirdly, pupil perceptions of a mastery-goal classroom learning structure are associated with a sense of belonging (Kumar, 2006), positive socio-emotional outcomes (Shim et al., 2013), less self-handicapping strategies (Ferguson and Dorman, 2003) and better achievement (Bennacer, 2000; Fast et al., 2010; Curby et al., 2011). Conversely, pupil perceptions of performance goal promotion and a competitive climate induce negative socio-emotional outcomes (Loukas and Murphy, 2007) and self-handicapping strategies (Ferguson and Dorman, 2003).

Even if non-exhaustive, the factors identified here allow us to draw more precise guidelines as to what we should aim for as we go forward in building IMT. These factors sketch out concrete outcomes rather than simply focusing on the ultimate and more abstract, distal goal of inclusion (e.g., achievement for all, stereotype-reduction and increased pro-social behavior). The next section continues to document this promising perspective. It focuses on how the measurements of these factors of inclusive classroom climate can help us develop the concrete building-blocks of IMT in classrooms and accurately evaluate their effects.

## 4. Inclusive classroom climate factors and the development of IMT

Studies identifying concrete IMT actions that actively support the inclusive factors of classroom climate are lacking (Tetler and Baltzer, 2011; De Vroey et al., 2016; Loreman, 2017). For example, in the mentioned search in scientific databases, only 10% of the 650 articles including “classroom climate” and “inclusion” were empirical studies investigating antecedents of classroom climate. These studies were moreover mostly based on correlational designs. The following section aims to show how being aware of the factors of inclusive classroom climate can help concretely develop IMT in the classrooms and guide the development of much-needed action research studies.

### 4.1. Factors of inclusive classroom climate perceived by students are proximal, sensitive, and pragmatic indicators of IMT

The factors of inclusive classroom climate are closer to day-to-day classroom life than the final inclusion-related outcomes, which need time to be changed. These factors are direct specific perceptions of classroom experiences. As such, they are more sensitive indicators of objective progress in IMT (Tetler and Baltzer, 2011). Sensitive indicators of IMT efficiency make it possible to detect even the smallest effects of modifications in IMT. That means that smaller samples and less time-consuming studies are required to obtain significant results and provide evidence-based practice guidelines. Sensitive indicators of efficient IMT are also essential to preserve teachers’ self confidence in their capacity to improve their practices. This is particularly important as teachers’ lack of self-confidence to teach in an inclusive manner has been well documented, along with the resulting impact on attitudes towards inclusive education (Desombre et al., 2019). Finally, the various validated measures of factors of classroom climate (for a review, Fraser, 2015) also provide evidence that the classroom climate approach is an appropriate and pragmatic way to identify effective IMT elements. Such measures can even be used in addition to final inclusive outcomes to test the expected mediation process and offer a more complete view of the effects of IMT modifications.

From another point of view, using students’ perceptions of classroom climate rather than teachers’ or observers’ reports is likely to be a more reliable way of developing IMT. Theoretically, students’ self-reports, compared to teachers’ and external observers’ reports, have the advantage of capturing the perceptions of the individuals whose behaviors are precisely the expected outcomes (Fraser, 1998). Students’ self-reports are also more precise because they are based on comparisons with other classroom climates that students have previously experienced throughout their schooling. They moreover result from all of the experiences that took place for them in the classroom, while external observers and even teachers only observed part of these experiences (Wang et al., 2020a). Evidence for this assumption can be found in studies comparing the relationships between inclusive outcomes and classroom climate measured according to these different sources. For example, in the study of Wang et al. (2020a), teachers’ reports did not significantly predict any student’ outcomes. Student reports had larger and more significant prediction effects when it came to student engagement and grades, whilst observers’ reports had smaller but better prediction effects on students’ long term achivement (i.e., standardized test scores after one year). Meta-analysis confirmed these results, as the source of reports of classroom climate (students, teachers, or observers) significantly moderated the strength of the relations between classroom climate and educational outcomes (Wang et al., 2020b). More precisely, only students’ reports of classroom climate were significantly associated with all outcomes assessed in the study (i.e., social competence, externalizing behaviors, socioemotional distress, engagement, and achievement). Additional evidence can be found in studies testing the effects of IMT modifications on both classroom climate and inclusive outcomes. For example, the study of Cheon et al. (2019) tested the longitudinal effects of a teacher training program on students’ social behaviors and measurements of inclusive classroom climate *via* students and observers’ reports. Results showed that students’ reports were as effective as observers’ reports in detecting changes in teaching style. But students’ reports of classroom climate were a better predictor of final inclusive outcomes than observers’ reports. Given that IMT needs to promote inclusive student outcomes to be effective, inclusive factors of classroom climate as perceived by students seem to be the best indicators of effective IMT. Students are key stakeholders to be consulted on issues that concern them, such as inclusion (Ainscow, 2007; Tetler and Baltzer, 2011).

Finally, classroom climate questionnaires are more pragmatic than videos, external observations, and techniques of naturalistic inquiry, ethnography, case study or interpretive research (see, Fraser, 1998). While recognizing that all methods have advantages and remain complementary, classroom climate questionnaires are particularly well adapted to school context. A last additional advantage is that classroom climate questionnaires can easily be used by teachers themselves if they want to evaluate their own practice and its evolution. Such procedures based on classroom climate questionnaires have already been developed and proved their efficiency in improving practices (see, Taylor et al., 1997; Fraser, 1998; Nelson et al., 2015; Moreu et al., 2021). Because they are easy to use, classroom climate questionnaires can also help to diagnose problems and develop responses before problems are grounded in classroom routines (Hoy and Woolfolk, 1990; MacNeil et al., 2009; Schweig et al., 2019).

To summarize, students’ self-reports of the listed inclusive factors of classroom climate are not only predictors of inclusive outcomes. They are also proximal, sensitive, and pragmatic indicators of IMT efficiency. They can help research to identify efficient elements of IMT, monitor progress, ensure that even small improvements are assessed, and assist professional growth (Schweig et al., 2019).

### 4.2. A multilevel multi-factor classroom climate measure to better identify effective IMT

IMT needs to be accessible to all students in the classroom. IMT aims to create a teaching experience that is shared by the entire classroom, rather than only focusing on marginalized students and trying to compensate their shortcomings. This universal accessibility is the core of the universal design for learning (Rose and Meyer, 2002), which led to promising results in promoting inclusion (Katz, 2013, 2015). This means that IMT improvements should result in improvements of student perceptions of the classroom climate for the large majority of students.

It follows that two methods can help to better document IMT improvement by means of students’ self-reports of classroom climate. First of all, the quality of IMT can be observed through the reduction of influence of students’ status (social and academic) in the classroom on their perception of classroom climate. Efficient IMT needs to increase engagement and learning for all students, and consequently independently from students’ status (Cohen, 1994; Lotan, 2006; Pescarmona, 2014; Lotan and Holthuis, 2021; Lotan, 2022). Measuring student status in addition to classroom climate provides the opportunity to document the decrease in the influence of student status on classroom climate which implies successful IMT implementation.

Second, IMT improvement can also be addressed through hierarchical linear modeling (HLM). HLM can distinguish the classroom level and the individual level of the students’ reports of classroom climate (Lüdtke et al., 2009; Marsh et al., 2012; Bardach et al., 2020). The common variance between students, at classroom level, constitutes an indicator of shared perceptions of the climate. Conversely, large residual differences between students in the same class, once shared agreement between students in the same class is controlled for, implies that there is significant diversity in perceptions of classroom climate. Efficient IMT is supposed to increase students’ reports of classroom climate and its inclusive consequences at the class level. Nevertheless, focusing on climate at the classroom level requires large sample studies, as the number of classes included in the analysis must be sufficient.

From another point of view, measuring several factors of the classroom climate at the same time offers a more effective measure of IMT. For example, cooperative pedagogy, often highlighted as a core element of IMT (e.g., Juvonen et al., 2019), is supposed to improve the student relationship aspect of classroom climate (Roseth et al., 2008). Nevertheless, cooperative pedagogy is also related to feelings of justice (Ghaith, 2003) and equity (Buchs and Maradan, 2021), and can improve learning, especially in a context of diversity (e.g., Falvey and Givner, 2005). To really catch the efficiency of cooperative pedagogy for inclusion hence requires measuring different factors of inclusive classroom climate. Similar conclusions can be reached when focusing on the inclusive effects of socioemotional learning programs, which primarily target the quality of relationships between students but can also impact factors of classroom climate associated with instruction (Durlak et al., 2011; Sklad et al., 2012). Moreover, hypothetical elements of IMT (e.g., Jigsaw cooperative method), often initially suggest fostering one factor of inclusive classroom climate (e.g., improving intergroup attitudes, Williams, 2004). But they may also undermine another factor (e.g., engagement, Cochon Drouet et al., 2022). Using a multi-factor measure of classroom climate helps to show that a single promising element of IMT has the potential to increase different factors of classroom climate, whilst maybe at the same time undermining others. Finally, the use of multi-factor measures of classroom climate is also a good way to diagnose specific difficulties in IMT and focus on one targeted factor (Moreu et al., 2021).

## 5. Conclusion

Despite the long-standing consensus on the need for inclusive education in educational systems around the world, mainstream schools are struggling to meet their two inclusion goals, i.e., promoting prosocial behaviors between diverse students and fostering engagement, learning and achievement for all students in a context of diversity. Recurrent problems such as bullying, school drop-out and ill-being are markers of, among other things, a lack of inclusion in the schools. This article helps address the discrepancy between the institutional will for inclusive schools and the reality of practices in the field. It reviews findings that show the added value of the classroom climate construct in developing more inclusive schools. According to these findings, the development of an inclusive classroom climate must be considered as the cornerstone of inclusive school building for three reasons.

Firstly, developing inclusive teaching in all classrooms is the core, yet also the main challenge of inclusive schools. Secondly, previous works clearly identify certain inclusive factors of classroom climate that are associated with the two inclusive objectives. Even if further work is needed to complete this picture, the factors reviewed constitute a preliminary definition of what characteristics a classroom climate needs in order to be inclusive. Thirdly, beyond the guideline that this definition of an inclusive classroom climate already represents, the classroom climate approach and associated validated measures can provide a hands-on way to develop IMT in the field. Indeed, measures of those listed factors, and especially students’ self-reports, are proximal, sensitive, and pragmatic indicators of effective IMT. A multilevel multi-factor classroom climate measure has the potential to document efficient IMT even more precisely. Moreover, such measures can also help teachers and education teams carry out an inclusive climate audit of their learning context (e.g., MacNeil et al., 2009), monitor their efforts in improving the situation (Nelson et al., 2015), and participate in their professional growth (Schweig et al., 2019; Moreu et al., 2021).

On this question of building inclusive schools, teachers have legitimate concerns when faced with the important professional transformation required by IMT in inclusive settings. Researchers are also struggling, as IMT is a practical challenge that requires being aware of the specific constraints that come with teaching. External bodies can draw up useful guidelines that ideally document associations between factors of classroom climate and inclusive outcomes. However, the concrete development of IMT in classrooms ultimately requires collaboration between teachers and researchers. The classroom climate construct constitutes an efficient tool to support these collaborations.

## Author contributions

The author confirms being the sole contributor of this work and has approved it for publication.

## Funding

Open access funding by University of Lausanne.

## Conflict of interest

The author declares that the research was conducted in the absence of any commercial or financial relationships that could be construed as a potential conflict of interest.

## Publisher’s note

All claims expressed in this article are solely those of the author and do not necessarily represent those of their affiliated organizations, or those of the publisher, the editors and the reviewers. Any product that may be evaluated in this article, or claim that may be made by its manufacturer, is not guaranteed or endorsed by the publisher.

## Footnotes

^1^This theoretical review aims to document why the classroom climate approach is a promising perspective to build more inclusive schools. In this perspective, empirical evidence that feeds each of the three steps of the argumentation was collected and organized. This review was firstly based on the analysis of empirical studies found in the systematic reviews and meta-analyses dealing with inclusive teaching or with the consequences and antecedents of classroom climate. It was completed with a search realized in January 2023 on *Sciences direct*, *Web of Sciences*, *ERIC*, *Pascal & Francis*, *APA PsyNET* and *Taylor & Francis* databases, for French or English articles including the keywords “inclusive” and “classroom climate.” This search led to 650 different articles that were analyzed and used to document each step of our argumentation.
